# NREM Parasomnias: Retrospective Analysis of Treatment Approaches and Comorbidities

**DOI:** 10.3390/clockssleep4030031

**Published:** 2022-08-16

**Authors:** Naina Limbekar, Jonathan Pham, Rohit Budhiraja, Sogol Javaheri, Lawrence J. Epstein, Salma Batool-Anwar, Milena Pavlova

**Affiliations:** Division of Sleep and Circadian Disorders, Brigham and Women’s Faulkner Hospital, 1153 Centre Street, Boston, MA 02130, USA

**Keywords:** NREM, parasomnias, confusional arousals, sleep walking, melatonin, benzodiazepines, obstructive sleep apnea, treatment

## Abstract

**Brief Summary:**

Non-rapid eye movement (NREM) parasomnias are relatively common among children and can often persist into adulthood, where they can pose as a clinical challenge:, the behaviors can result in injury or have negative impacts on functioning and quality of life, thus necessitating treatment, but the choice of treatment is complicated by the lack of evidence—based guidelines, as well as potential side effects. The aim of this retrospective analysis is to examine the most frequently used treatment strategies and pharmaceuticals for NREM parasomnias and evaluate perceived outcomes based on the patient’s subjective reports of the frequency and severity of symptoms.

**Abstract:**

The aim of this retrospective analysis is to determine the most frequently prescribed medications for the treatment of NREM parasomnias and evaluate reported outcomes. We performed a retrospective chart review of all patients with NREM parasomnia diagnosed within Brigham and Women’s Hospital (BWH) clinics examining the date of diagnosis, date of starting therapy, comorbidities, type of medication prescribed, and the reported change in symptoms or side effects at follow-up visits. From 2012 to 2019, 110 patients (59 females, 51 male) at BWH clinics received a diagnosis of NREM parasomnia, including sleepwalking and night terrors. The mean age was 44. Comorbidities included obstructive sleep apnea (OSA) (46%), periodic limb movement syndrome (PLMS) (13%), insomnia (19%), Restless leg syndrome (RLS) (9%), epilepsy (4%), and REM behavior disorder (RBD) (9%). Initial treatment strategies include behavioral and safety counseling only (34%), pharmacological treatment (29%), treatment of any comorbidity (28%), and combined treatment of any of the above (9%). Improvement was reported with: treatment of OSA (*n* = 23 52% reported improvement), melatonin (*n* = 8, improvement reported by 88%.,benzodiazepine (*n* = 7, improvement reported by 57%). Treating comorbid conditions is a frequent treatment strategy, often associated with symptom improvement. The pharmacologic treatment most commonly included melatonin and benzodiazepines. Comprehensive management should include behavioral and safety recommendations, assessment of comorbid conditions, and individually tailored pharmaceutical treatment.

## 1. Introduction

NREM parasomnias encompass diverse and heterogeneous phenomena occurring during sleep that tend to be underdiagnosed and consequently undertreated due to a lack of evidence-based treatment guidelines. “Parasomnia” is a term that was first coined by French researcher Henri Roger in 1932 and was derived from the Greek word “para”, meaning “along the side of”, and the Latin term “somnnus”, which means “sleep” [1]. The pathophysiology of parasomnias is hypothesized to be central nervous system (CNS) activation and intrusion of wakefulness into either NREM or rapid eye movement (REM) sleep, resulting in non-volitional motor, autonomic, or emotional activity that is typically undesirable, sometimes even hazardous. This activity is usually followed by an altered perception of the environment, incomplete responsiveness to external stimuli, and often retrograde amnesia [2]. NREM parasomnias, also called disorders of arousal, tend to occur out of slow wave or stage N3 sleep with a predominance in the first third of the night. The most common NREM phenotypes include sleepwalking, sleep talking, sleep terrors, sleep-related eating, and confusional arousal [3]. Although typically believed to be a childhood disorder, they can persist into adulthood, where lifetime and current prevalence presenting to clinics includes sleep talking with 66.8% and 17.7%, confusional arousal with 18.5% and 6.9%, sleep terror with 10.4% and 2.7%, sexual acts during sleep with 7.1% and 2.7%, and sleep-related eating with 4.5% and 2.2% [4], respectively. Additionally, a small but notable population of patients may also be at risk of injury, where current prevalence indicates that 4.3% of patients with parasomnias are injured during sleep and 0.9% injure somebody else during sleep [4]. Disorders of arousal that persist into adulthood are not characterized sufficiently in the literature, and yet these adult behaviors are often more dangerous (i.e., may result in injury) or have negative impacts on the individual’s functioning and quality of life, resulting in significant social and forensic implications as compared to those occurring in childhood [5]. Therefore, the effectiveness of treatment of these conditions is relevant.

Although the precise mechanisms leading to disorders of arousal are yet to be determined, there are a number of predisposing, priming, and perpetuating factors that have been identified [6]. Predisposing factors include a genetic tendency, as patients often report a family history and the presence of sleepwalking or a related disorder in a first degree relative increases the chances of developing this disorder by a factor of 10 [7]. HLA gene DQB1 is present in 35% of sleepwalkers as compared to 13.3% of normal controls [8]. Genetic testing can often be difficult to obtain and has unclear clinical utility. Priming factors such as sleep deprivation, hypnotic drugs, and stress are thought to increase the proportion of stage three sleep, which provides more opportunity to give rise to these undesirable events [6]. Precipitating factors are those that increase the chances for arousal from slow wave sleep and may include sleep-disordered breathing (SDB) and periodic limb movements in sleep (PLMS) [6].

Treatment strategies for NREM parasomnias often include behavioral and standard safety recommendations, the treatment of comorbid conditions (OSA, PLMS, restless legs syndrome (RLS), insomnia, migraines, seizures), or the primary treatment of the parasomnia itself with pharmacotherapy, including melatonin, benzodiazepines, and tricyclic antidepressants. However, the decision whether and how to initiate treatment has not been evaluated systematically for efficacy and currently there are no specific controlled treatment trials for pharmacological options. The aim of this study is to describe and assess the effectiveness of clinically chosen treatments prescribed to a cohort of patients with a diagnosis of NREM parasomnias with or without coexisting comorbid sleep, neurological and psychiatric disorders to aid in the future development of more standardized treatment strategies.

## 2. Results

**Figure 1:**
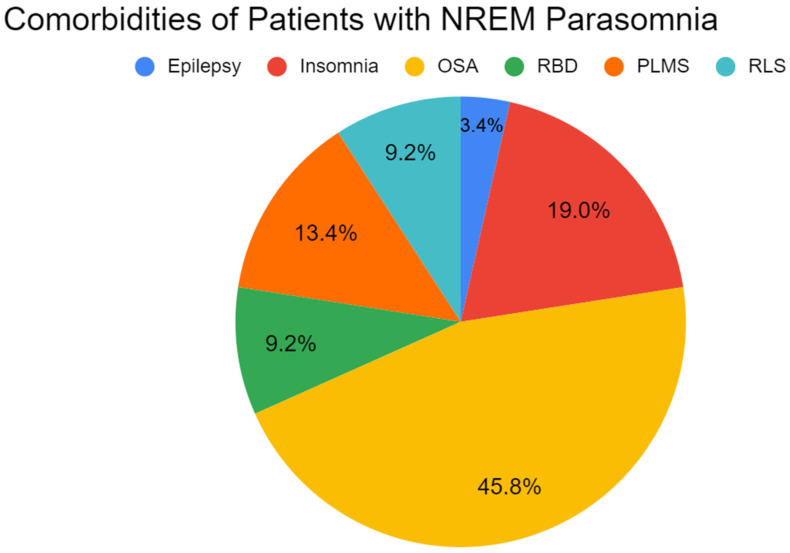
Proportions of comrbidites of patients with NREM parasonmia.

From 2012 to 2019, 110 patients (59 females, 51 male) at BWH clinics received a diagnosis of NREM parasomnia. Phenotypes included sleep walking (35%), sleep talking (20%), night terrors (14%), violent movements including kicking and screaming (19%), dream enactment (7%), and sleep eating (5%). The average age of onset for males was 47 years and for females it was 42 years. After stratifying by gender, there were no statistically significant differences between the two groups regarding the parasomnia phenotypes, age of onset, or comorbidities (data not shown). Figure 1 depicts comorbidities including obstructive sleep apnea (OSA) (46%), PLMS (13%), insomnia (19%), RLS (9%), REM behavior disorder (RBD) (9%) (considered parasomnia overlap), and epilepsy (4%)**.** Table 1 depicts pertinent concurrent medications including clonazepam (29%), lorazepam (29%), temazepam (7%), alprazolam (7%), imipramine (3%), amitriptyline (7%), mirtazapine (4%), and melatonin (14%).

**Treatment Strategies:** Initial treatment strategies included behavioral and safety counseling only (34%), pharmacological treatment (29%), treatment of any comorbidity (28%), combined treatment of any of the above (9%). The overall success rate of the combined treatments was 60%.

**Behavioral and Safety Precautions:** Of the patients who received this treatment strategy, it was effective in 5%, not effective in 13%, and 82% of patients were lost in the follow-up process.

**Figure 2:**
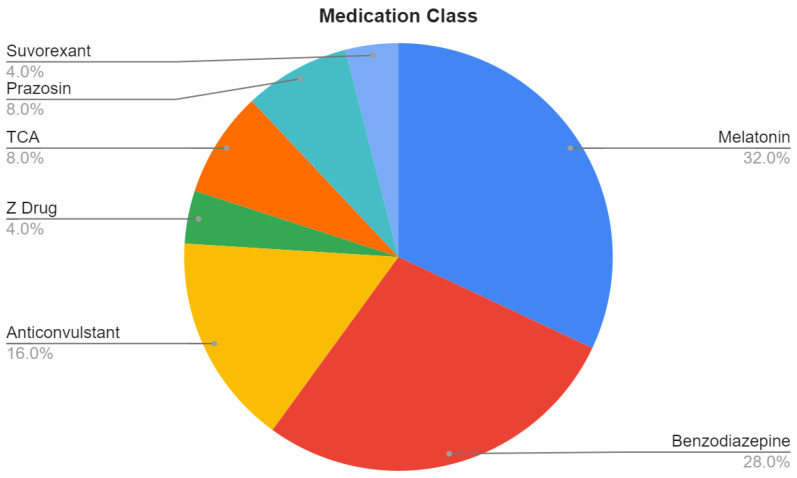
Proportions of medications taken by subjects.

**Pharmacological Treatment: **The most common medication classes used to treat the NREM parasomnias were melatonin (32%), benzodiazepines (28%), anticonvulsants (16%), tricyclic antidepressants (8%), prazosin (8%), Z-drugs/nonbenzodiazepine drugs (4%), and suvorexant (4%), as depicted in Figure 2. Melatonin treatment (*n* = 8) was reported effective by 88% of patients. The most used benzodiazepine was clonazepam (*n* = 5), and 40% of patients reported improvement, another 40%—no effect, and 20% were lost to follow up. Fewer than five individuals used prazosin, topiramate, imipramine, suvorexant, or gabapentin.

**Treatment of Comorbidity:** The most treated comorbidity was OSA (*n* = 23), and this treatment was associated with reduced parasomnia behaviors in 52% of patients. The treatment of OSA involved positive airway pressure therapy (PAP). Improvement was also reported after treatment of migraine (*n* = 2–50% of patients reported improvement), and RLS (*n* = 4), improvement reported by 50% of patients. Patients who were treated for epilepsy, narcolepsy, insomnia, and seizure did not have follow up records available. Overall, 30% of patients who underwent treatment of their comorbidity as a strategy were lost in the follow-up process.

**Combination Treatment Strategies:** The most common combination treatment strategy was behavior and safety recommendations and treatment of a comorbidity (*n* = 4), and this was reported effective in 50% of patients (Table 2). The combination of pharmacological treatment and treatment of comorbidity (*n* = 3) was reported to be effective in 33% of patients (Table 2). Behavior and safety recommendations combined with pharmacological treatment (*n* = 3) was reported as effective in 67% of patients (Table 2).

## 3. Discussion

This study reports a sizable cohort of patients treated for NREM parasomnias of variable phenotypes given the relative rarity of this sleep pathology in the adult population. Treatment strategies started off with behavioral and safety recommendations, followed by various pharmacological treatments, as well as the treatment of comorbid conditions as precipitating factors for the development of NREM Parasomnias.

The efficacy of behavior and sleep hygiene recommendations is unclear, as only 18% of patients treated with this strategy alone returned for a follow-up. Nonetheless, this treatment strategy, which revolves around minimizing precipitating factors including avoiding sleep deprivation and minimizing alcohol to reduce sleep fragmentation, should provide less opportunity for the occurrence of NREM parasomnias with minimal to no harm [6]. Regardless of the phenotype of NREM parasomnias, patient education regarding the use of bed alarms, avoidance of forced awakenings, and locking of windows/doors may be necessary to avoid suffering debilitating injuries. Standard sleep hygiene pamphlets are widely available including by the American Academy of Sleep Medicine.

This study corroborates some of the previously described literature regarding commonly prescribed pharmacological therapy for NREM parasomnias. Melatonin was reported to be the most efficacious of all medications, with 88% of patients reporting clinical symptom improvement at follow-up visits. Melatonin tends to be widely available and confers little harm and may serve as an initial pharmacological treatment strategy for patients with various phenotypes. The improvement seen in this study, in addition to prior literature, may suggest an underlying circadian misalignment as a potential component to the development of NREM parasomnias. Prior work implicates a potential circadian pathogenesis for NREM parasomnias. NREM parasomnias have been more prevalent in nurses working alternating night–day shifts as compared to those with daytime shifts only [9]. Further research exploring whether underlying potential circadian disorders, such as delayed sleep wake phase disorder give rise to the development of NREM parasomnias is needed and could potentially provide individually tailored treatment plans without need for escalating treatment with potentially more harmful pharmacological intervention. Finally, another mechanism by which melatonin may have been effective is via partial treatment of sleep deprivation or insomnia, which could function as a precipitating factor for these NREM events.

The commonly used strategy of treatment with benzodiazepines was also observed in our patient population. The most frequently used was clonazepam which was reported to be effective in 40% of those treated. The use of lorazepam was 100% effective in the small number of patients treated. The proposed mechanism of benzodiazepines in the treatment of these events is by reducing slow wave sleep, which NREM parasomnias generally arise from. Another possible mechanism is by increasing the arousal threshold, decreasing arousal and stabilizing sleep. Benzodiazepines may be helpful as a treatment strategy especially in patients with frequent events and events that may result in physical injury or harm (i.e., violent thrashing, sleepwalking), as well as considered for those with comorbid anxiety or post traumatic stress disorder (PTSD), who are thought to have a low arousal threshold. Appropriate caution in the use of benzodiazepines is recommended for patients with concurrent disorders including advanced age, fall risks, abuse potential, and sleep-disordered breathing, as these outcomes could worsen respiratory depression and may result in the worsening of apnea and NREM parasomnia events.

This study highlights the importance and consideration of the treatment of comorbid sleep disorders that may serve as precipitating factors for the development of NREM parasomnias. OSA was prevalent in 46% of patients diagnosed with NREM parasomnia and treatment with PAP alone was effective in controlling symptoms in 52% of patients, suggesting a valid treatment strategy. The successful treatment of nocturnal respiratory events with PAP results in more consolidated sleep with less opportunity for arousals and thus parasomnia events. In addition, the treatment of OSA with PAP therapy has been shown to improve symptoms of insomnia and thus may provide a secondary mechanism in which this strategy is effective for reducing NREM events [9].

**Limitations:** This study has multiple limitations, including the retrospective nature of the study design. As a result, the efficacy of treatment outcomes has been solely based on the patient’s subjective reports of their symptoms, which introduces the possibility of recall bias. This may be further compounded by the fact that patients generally tend to be amnestic of their NREM events. It is possible that patients may have overreported their improvement potentially due to a placebo effect of office visits with a sleep specialist. Additionally, many individuals had no available follow-up information. Furthermore, the population is heterogeneous and many had comorbidities, thereby including likely parasomnia overlap (since RBD and NREM parasomnia events were reported in the clinical history), which may also have influenced reported efficacy. Among those individuals, melatonin may have improved the events that arise from REM sleep. From the existing data, there is no possibility to test whether this is the reason for reported improvement in symptoms. Another significant limitation lies in the fact that there was no objective confirmation of medication compliance thus resulting in greater results of efficacy of various pharmacological treatments. However, results were more robust for the comorbid OSA treatment group, as PAP compliance was assessed at office visits. An additional limitation lies in a small sample size of the patient cohort, particularly for the sub-analyses regarding pharmacological treatment. Data were especially limited for the samples of patients treated with prazosin, imipramine, and topiramate, and thus no comments can be made on their respective efficacy.

**Strengths:** The primary strength of this study lies in the large and heterogeneous patient cohort for an unusual adult disorder. By studying a sleep clinic cohort, we were also able to study the contributions of common comorbid sleep pathologies.

## 4. Methods

We retrospectively identified patients with a clinical diagnosis of NREM parasomnia, irrespective of other concomitant sleep disorders, following a consultation with a sleep physician at all sleep disorder clinics at Brigham and Women’s Hospital (BWH) and Brigham and Women’s Faulkner Hospital (BWFH) over a period of seven years (from 2012 to 2019) using the following ICD−10 codes: G47.51, F51.3, F51.4, F51.5, and G47.59. Appropriate approval from the institutional review board on human research was obtained.

The differential diagnosis of nocturnal seizures versus NREM parasomnia is a common consideration as they commonly have a typical presentation. For this reason, we have an established a practice to use extended EEG data when patients with NREM parasomnia are evaluated in the clinical sleep laboratory [10]. We consider this an important component of the evaluation of nocturnal events, and this is the standard for evaluation in our clinic. We examined the date of diagnosis, demographic features, comorbid conditions, date of starting therapy, type of medication used, and changes in symptoms at the individual’s follow-up visit, as well as any side effects observed. We then analyzed the efficacy of treatment outcomes. Efficacy of treatment was determined by the patient’s subjective reports of either complete resolution or satisfactory control of symptoms without experiencing significant side effects from treatment strategies. In addition, all patients received standard safety recommendations and good sleep education prior to the initiation of any other treatment. Safety recommendations included removing knives and sharp objects in the bedroom, implementing bed padding or bed railing, and locking windows and doors. Good sleep education involved explaining and offering a pamphlet of recommendations compiled by sleep medicine experts at Brigham and Women’s Hospital, which included but was not limited to regularizing sleep routines, minimizing disruptions during sleep, and avoiding caffeine/alcohol/smoking before bedtime.

## 5. Conclusions

Patient education regarding safety and behavioral sleep hygiene recommendations highlighting the importance of avoiding sleep deprivation is a major component of treating parasomnias. Next, the evaluation and treatment of comorbid sleep pathologies, such as sleep-disordered breathing, may also confer significant benefits in outcomes. After the above facets are addressed, an individually tailored pharmacological intervention may be considered.

## Figures and Tables

**Table 1:** Frequencies and proportions of pertinent concurrent medications.

*Pertinent Concurrent Medications*		
Clonazepam	30	29.70%
Lorazepam	29	28.71%
Temazepam	7	6.93%
Alprazolam	7	6.93%
Other Benzo ending in ZEPAM	0	0.00%
Imipramine	3	2.97%
Amitriptyline	7	6.93%
Mirtazapine	4	3.96%
Melatonin	14	13.86%

Total number of medications used: 101.

**Table 2:** Effectiveness of combinations of treatment strategies.

Combination of Treatment Strategies				
Combination	Effective	Not Effective	Lost	Total
Pharmacological + Comorbidity	1	1	1	3
Behavioral & Safety + Comorbidity	2	1	1	4
Behavior & Safety + Pharmacological	2	0	1	3
Total	5	2	3	10

Total number of combinations of treatment strategies used: 10.

## Data Availability

The data is kept within the BWH encrypted computers and can be available if needed within 7 years of the study after which they will have to be destroyed for the purposes of patient confidentiality.

## Abbreviations

BDZ	Benzodiazepines
BWH	Brigham and Women’s Hospital
BWFH	Brigham and Women’s Faulkner Hospital
CNS	Central Nervous System
NREM	Non-Rapid Eye Movement
OSA	Obstructive Sleep Apnea
PAP	Positive Airway Pressure
PLMS	Periodic Limb Movements in Sleep
PTSD	Post-Traumatic Stress Disorder
RBD	Rapid Eye Movement Behavior Disorder
REM	Rapid Eye Movement
RLS	Restless Leg Syndrome
SDB	Sleep-Disordered Breathing
TCA	Tricyclic Antidepressant
